# Loss of slit protein nephrin is associated with reduced antioxidant superoxide dismutase expression in podocytes shed from women with preeclampsia

**DOI:** 10.14814/phy2.13785

**Published:** 2018-07-06

**Authors:** Yuping Wang, Shuang Zhao, Yang Gu, David F. Lewis

**Affiliations:** ^1^ Department of Obstetrics and Gynecology Louisiana State University Health Sciences Center Shreveport Louisiana

**Keywords:** CuZn‐SOD, kidney, nephrin, oxidative stress, podocyte injury, preeclampsia

## Abstract

Recent findings of podocyte shedding/podocyturia highlight the central significance of podocyte injury in preeclampsia, a hypertensive disorder unique to human pregnancy. To test the hypothesis that oxidative stress contributes to kidney podocyte injury in preeclampsia, we specifically examined expression and distribution of antioxidant CuZn‐SOD with nephrin and podoplanin in shed podocytes from women with preeclampsia. Human podocyte AB 8/13 cells served as control. We found that CuZn‐SOD was localized at the front/outreach region of nephrin at the cell periphery (foot process areas) in control podocytes and expression of CuZn‐SOD, nephrin, and podoplanin were all dislocated or lost in shed podocytes from preeclamptic patients. We further tested oxidative stress‐induced nephrin shedding in podocytes, in which AB 8/13 podocytes were cultured under lowered oxygen condition (2%O_2_) or treated with hypoxic mimicking agent cobalt chloride. Our results showed that reduced nephrin and podoplanin expression were associated with downregulation of CuZn‐SOD expression in podocytes when cells were cultured under lowered oxygen or hypoxic conditions. Nephrin shed in urinary specimen from preeclamptic women was also determined by immunoprecipitation/immunoblotting. The molecular sizes of nephrin that corresponded to that were lost when cells were cultured under hypoxic conditions. We concluded that increased oxidative stress plays a significant role in inducing podocyte protein shedding in preeclampsia.

## Introduction

Proteinuria and glomerular endotheliosis constitute the kidney lesion in preeclampsia, a hypertensive and multi‐system disorder in human pregnancy. In addition to the classically coined endotheliosis (Spargo et al. 1959), podocyte, glomerular visceral epithelial cell, injury has recently been merged as an important kidney glomerular lesion in preeclampsia. Evidence of podocyte injury includes reduced podocyte‐specific protein nephrin and synaptopodin expression seen in kidney autopsy or biopsy specimen from women who had preeclampsia (Garovic et al. 2007a; Zhao et al. 2009), and shed podocytes found in urinary specimen from pregnant women complicated with preeclampsia (Garovic et al. 2007b; Aita et al. 2009; Zhao et al. 2011). Our recent studies further revealed that not only is podocyte shedding associated with severity of the disease (Zhao et al. 2011), but also that urine levels of nephrin and podocalyxin are correlated with the amount of proteinuria in women with preeclampsia (Wang et al. 2012, 2015). These findings clearly indicate that podocyte injury occurs in preeclampsia. Proteinuria is a signature of podocyte injury. Therefore, there is no doubt that podocyte shedding and podocyte‐specific protein shedding contribute to kidney pathology in this unique pregnancy disorder.

Nephrin is a specific podocyte slit protein and is proposed to be the backbone of the slit diaphragm (Tryggvason et al. 2006). Genetic mutation of the nephrin gene during development has been demonstrated to be responsible for the congenital nephrotic syndrome of Finnish type (Kestilä et al. 1998). In preeclampsia, nephrin expression is reduced in podocytes (Garovic et al. 2007a; Zhao et al. 2009) and loss of the major glycoprotein nephrin from slit diaphragms is responsible for increased plasma protein leakage in preeclampsia. Although it is speculated that altered angiogenic factor VEGF and its soluble receptor sFlt‐1 may interrupt kidney glomerular function in preeclampsia (Karumanchi and Lindheimer 2007), virtually little is known about the cause of podocyte injury in this pregnancy disorder.

We previously reported that reduced nephrin and podoplanin expression is associated with increased nitrotyrosine staining and reduced superoxide dismutase expression in kidney biopsy tissue sections from women who had preeclampsia (Zhao et al. 2009). Nitrotyrosine is a maker of increased oxidative stress and is formed when a protein molecule is nitrated by peroxynitrite, which is generated via the reaction of superoxide radical with free radical nitric oxide. Superoxide dismutase is an antioxidant enzyme to dismutate superoxide radicals within cells. Thus, enhanced nitrotyrosine staining and reduced superoxide dismutase expression point out increased oxidative stress and/or increased superoxide generation in kidneys in preeclampsia. Podocytes express reduced form of nicotinamide adenine dinucleotide (NADH), which is the primary source for superoxide generation (Greiber et al. 1998). Therefore, sufficient antioxidant activity and capacity could be critical to protect podocytes from oxidative insult. CuZn‐superoxide dismutase (CuZn‐SOD) is one of the three superoxide dismutases, which are responsible for destroying free superoxide radicals and protecting cells from superoxide damage. In this study, we tested our hypothesis that oxidative stress induces podocyte injury and podocyte protein shedding in preeclampsia. We examined CuZn–SOD expression and distribution associated with nephrin and podoplanin expression in shed podocytes from women with preeclampsia. Effects of hypoxia/oxidative stress on podocyte nephrin and podoplanin expression were also assessed.

## Materials and Methods

### Chemicals and reagents

Medium RPMI 1640 was purchased from GIBCO, Carlsbad, CA; Antibiotic‐antimycotic solution, insulin‐transferrin‐selenite (ITS) liquid media supplement, and protein‐A immunoprecipitation kit were from Sigma‐Aldrich, St. Louis, MO; Fetal bovine serum (FBS) was from Atlantic Biologicals (Flowery Branch, GA); Cobalt chloride (CoCl_2_, 4532‐02) was from Mallinckrodt Chemicals, Phillipsburg, NJ; Antibody for Wilm's tumor suppressor gene 1 (WT‐1, CAN‐R9‐56‐2) was purchased from Epitomics, Burlingame, CA; Antibody against podocin (ab65291) was from Abcam, Cambridge, MA; and antibodies against podoplanin (FL‐162, sc‐134482) and CuZn‐SOD (ab52950) were from Santa Cruz, San Diego, CA; Cy‐3‐conjugated secondary antibody was from Jackson Immunotech Lab, Westgrove, PA; and Alex488‐conjugated secondary antibody was from Molecular Probe/Invitrogen, Carlsbad, CA; Anti‐nephrin (ab58968) from Abcam was used for immunofluorescent staining and Anti‐nephrin (N‐16, sc‐32532) from Santa Cruz was used for immunoprecipitation/immunoblotting. Vectashield Mounting Medium with DAPI was purchased from Vector Lab Inc., Burlingame, CA. All other chemicals were from Sigma unless otherwise noted.

### Urine specimen collection

Normal and preeclamptic pregnant women were recruited when they were admitted to Labor and Delivery at the University Health Hospital in Shreveport, Louisiana in affiliation with Louisiana State University Health Sciences Center in Shreveport (LSUHSC‐Sh). The study was approved by the Institutional Review Board (IRB) for human research at LSUHSC‐Sh. Normal pregnancy was defined as an uneventful pregnancy with blood pressure <140/90 mmHg and absence of proteinuria. Preeclampsia was defined as elevated blood pressure (>140/90 mmHg) on two separate occasions at least 6 h apart after 20 weeks of gestation, proteinuria of more than 300 mg in a 24‐h urine, or more than 1+ on dipstick of random urine samples. Urine samples were collected after informed consent was obtained. A total of 20 patients were recruited in this study, 10 normal and 10 preeclamptic pregnancies. The clinical information of normal and preeclamptic pregnant women, including maternal age, racial status, blood pressure, body mass index (BMI), gestational age at urine sample collection and delivery, and delivery mode, is shown in Table 1. None of the study subjects had signs of infection. Smokers and pregnancies complicated by nephrotic syndrome and/or diabetes were excluded from the study.

**Table 1:** Demographic data for normal and preeclamptic pregnant women

	Normal pregnancy (*n* = 10)	Preeclampsia (*n* = 10)	*P* value
Maternal age (years)	26 ± 5	27 ± 4	NS
Racial status			
White	2	2	NA
Black	7	8	NA
Other	1	0	NA
Gestational age (weeks)			
Urine collection	38 ± 2	32 ± 4	*P* < 0.01
At delivery	38 ± 2	33 ± 3	*P* < 0.01
Blood pressure (mmHg)			
Systolic	119 ± 12	163 ± 9	*P* < 0.01
Diastolic	68 ± 8	94 ± 12	*P* < 0.01
Proteinuria	(‐)	1+ ‐ 4+	*P* < 0.01
BMI	31 ± 6	35 ± 9	*P* = 0.26
Delivery mode			
Vaginal	2	1	NA
C‐section	8	9	NA

Data are presented as mean ± SD.

BMI: body mass index; NS: not significant; NA: not analyzed; Proteinuria was expressed as dipstick.

### Podocyte extraction and culture

Freshly obtained urine specimens were centrifuged at 450 *g* at 4°C for 5 min. Cell pellets were suspended and washed twice with medium RPMI 1640. For culture, cell pellets were incubated with medium RPMI 1640 supplemented with 10% FBS, antibiotic‐antimycotic solution, and insulin‐transferrin‐selenite (ITS) liquid media supplement as previously described (Saleem et al. 2002). Cell suspension was seeded into fibronectin‐coated 6 well/plate for total cellular protein collection or 24 well/plate on cover slips for cell morphology study. Cells were incubated at 37°C with 5%CO_2_ and air. The medium was changed every other day. Immunofluorescent staining was performed between 10 and 15 days after seeding.

### Culture of human podocyte cell line AB 8/13 cells

AB 8/13 cells (conditionally immortalized human podocytes) are an established human podocyte cell line, and a kind gift from Dr. Moin A. Saleem at University of Bristol, Bristol, UK. AB 8/13 cells were produced by transfection of both tsSV40 and human telomerase (hTert) to primary human podocytes (O'Hare et al. 2001). AB 8/13 cells were cultured with medium RPMI 1640 supplemented with 10% FBS, antibiotics, and ITS liquid media supplement as described previously. These cells proliferate under the permissive temperature of 33°C driven by the temperature – sensitive SV40 gene. Upon thermoswitching to the nonpermissive temperature at 37°C, SV40‐T gene is inactivated and the cells begin to differentiate. After being cultured for 10–14 days, differentiated AB 8/13 cells express slit diaphragm – specific markers such as nephrin and synaptopodin, etc. (Saleem et al. 2002). Differentiated AB 8/13 cells served as control to shed podocytes from preeclampsia.

### Induction of oxidative stress in podocytes

Oxidative stress was induced by two conditions on differentiated AB 8/13 podocytes: (1) cells were cultured under reduced oxygen condition, 2%O_2_/5%CO_2_ and balanced with 93%N_2_ for 24 h, and (2) cells were treated with cobalt chloride at 100 *μ*mol/L for 2 h. CoCl_2_ is a hypoxic mimic reagent, which can initiate oxygen‐sensing signal transduction pathway by upregulation and stabilization of HIF1*α* expression (Goldberg and Schneider 1994; Maxwell et al. 1999; Dai et al. 2012). Thus, CoCl_2_ has been widely used as a hypoxic mimic reagent to induce oxidative stress both in vivo and in vitro experiments (Goldberg and Schneider 1994; Ma et al. 2011).

### Immunofluorescent staining

Expression and distribution of nephrin, podoplanin, and CuZn‐SOD were examined by either single or dual immunofluorescent staining. Briefly, cells grown on glass cover slips were fixed with 2% paraformaldehyde in 4% sucrose for 8 min at room temperature, followed by washing 3× with 1% bovine serum albumin (BSA) in phosphate‐buffered saline (PBS), each for 5 min. After blocking, cover slips were incubated with primary antibodies, including WT‐1, podocin, nephrin, podoplanin, and CuZn‐SOD, followed by matched secondary antibody. Secondary antibody was either Cy‐3‐conjugated or Alex488‐conjugated. After staining, cover slips were mounted on glass slides with Vectashield Mounting Medium with DAPI. Slides were reviewed under a fluorescent microscope (Olympus IX‐71, Japan). Images were captured with a digital camera linked to a computer with imaging software PictureFrame (Uptronics Inc. Sunnyvale, CA). Images were also captured by Apotome Observer (Carl Zeiss, Inc. Germany) and Z‐stacking images were reconstructed with Axiovision software (Carl Zeiss, Inc.).

### Nephrin and CuZn‐SOD expression

Protein expression for nephrin and CuZn‐SOD was also determined by Western blot. For podocyte expression, an aliquot of total podocyte cellular protein 10 *μ*g per sample was run on a Mini‐cell protein‐3 gel running system (Bio‐Rad, Hercules, CA) and transferred to Hybond‐protein transfer membranes (Amersham Corp, Arlington Heights, Ill). The membranes were probed with primary antibody to nephrin or CuZn‐SOD overnight and then by horseradish‐peroxidase linked secondary antibody the following day. Bands were visualized with an enhanced chemiluminescent detection kit (Amersham Corp.). *β*‐actin expression was determined to ensure an equal loading of the samples.

Nephrin expression was also determined by immunoprecipitation/immunoblotting in urinary specimen from normal and preeclamptic pregnancies. Protein‐A immunoprecipitation kit (Sigma) was used following the manufacturer's instructions. Briefly, an aliquot of 10 mL of urinary specimen was centrifuged at 1850 *g* for 10 min and then the clear phase of urinary specimen was precipitated with nephrin‐labeled protein‐A beads. The precipitated protein was run on SDS‐PAGE and transferred to a nitrocellulose membrane, which was probed with nephrin antibody followed with the secondary antibody. The bound antibody was visualized with an enhanced chemiluminescent (ECL) deletion kit (Amersham Corp).

## Results

### Podocytes were found in urine specimen from preeclampsia

**Figure 1:**
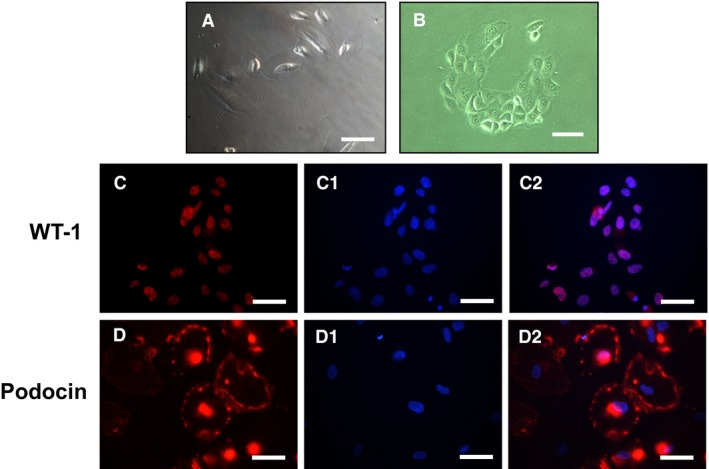
Urine cells grown in culture and cells stained with WT‐1 and podocin. (a) and (b) representative urine cells grown in culture, bar = 100 *μ*m; (c) and (d) cells stained with podocyte markers WT‐1 (c) and podocin (d); c1 and d1 are DAPI staining and c2 and d2 are merged of c and c1, and d and d1, respectively. B = 50 *μ*m.

Urine specimen was obtained from 20 pregnant women, 10 from normal and 10 from preeclamptic pregnancies. After 10 days of culture, podocytes were grown in all cultures from preeclamptic, but not in normal, urine specimens. Podocytes were identified by positive expression of podocyte markers including Wilm's tumor suppressor gene 1 (WT‐1) and podocin. Figure 1 shows cells grown in culture and cells stained with WT‐1 and podocin, which indicates shed cells in urine specimens from women with preeclampsia are podocytes.

### Reduced expression of nephrin, podoplanin, and CuZn‐SOD in shed podocytes from women with preeclampsia

**Figure 2:**
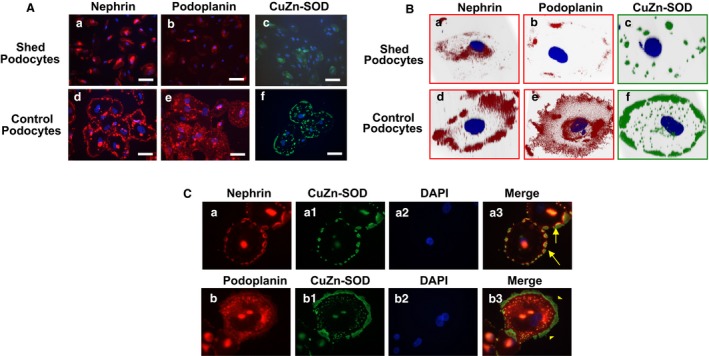
Expression of nephrin, podoplanin, and CuZn‐SOD in control podocytes and in shed podocytes from women with preeclampsia. (A) Comparison of nephrin, podoplanin, and CuZn‐SOD expression between shed podocytes from preeclampsia and control podocytes. a–c: shed podocytes; d–f: control podocytes. Nephrin, podoplanin, and CuZn‐SOD are barely detectable on shed podocytes from preeclamptic patients. Bar = 100 *μ*m. (B) Three‐dimensional reconstruction of *Z*‐stacking images of nephrin, podoplanin, and CuZn‐SOD on podocytes. a–c: shed podocytes from preeclampsia; d–f: control podocytes. In control cells, both nephrin and CuZn‐SOD are localized at cell periphery (d and f), the presumptive foot process area; and podoplanin is expressed throughout the cell surface (e). Loss or reduced nephrin, podoplanin, and CuZn‐SOD expression are seen in shed podocytes from preeclampsia (A a–c; and B a–c, respectively). (C) Dual immunofluorescent staining of nephrin/CuZn‐SOD and podoplanin/CuZn‐SOD in control podocytes. Both nephrin and podoplanin show close association with CuZn‐SOD at the cell periphery region. CuZn‐SOD looks like a “stake” holding nephrin in place (arrow in a3). Similar pattern is also noticed for podoplanin and CuZn‐SOD, with the associated setting more pronounced at the cell periphery. “a” series: nephrin (a), CuZn‐SOD (a1), DAPI (a2), and merge (a3); and “b” series: podoplanin (b), CuZn‐SOD (b1), DAPI (b2), and merge (b3).

To determine if altered nephrin, podoplanin, and CuZn‐SOD expression is present in shed podocytes from preeclamptic patients, expression and distribution of nephrin, podoplanin, and CuZn‐SOD was examined in shed podocytes from preeclamptic pregnant women and AB 8/13 podocytes. Figure 2 shows representative images of nephrin, podoplanin, and CuZn‐SOD expression in shed podocytes in comparison to AB 8/13 podocytes. Figure 2A shows images at a low magnification captured by a fluorescent microscope and Figure 2B shows images at a high magnification captured by Apotome Observer. Consistent results were obtained. In control AB 8/13 cells, nephrin expression is mainly localized and distributed at the cell periphery in a punctuated pattern, corresponding to the putative foot process areas, and podoplanin is expressed on the cell surface. Interestingly, similar to nephrin, CuZn‐SOD is also primarily localized in the cell periphery in a punctuated or continuous pattern in control differentiated podocytes, while in shed podocytes from women with preeclampsia, nephrin was undetectable at the cell periphery but seen in the cytosol. Similarly, expression of podoplanin and CuZn‐SOD were also reduced or lost in shed podocytes from preeclampsia compared with AB 8/13 differentiated podocytes (Fig. 2A and B).

### CuZn‐SOD is localized at the cell periphery area and closely related to nephrin expression in differentiated podocytes

Since both nephrin and CuZn‐SOD are localized at the cell periphery in differentiated control podocytes, we examined the association of CuZn‐SOD with nephrin and podoplanin in podocytes by dual immunofluorescent staining. Again, our results showed that CuZn‐SOD is mainly expressed in the cell periphery in a punctuated or continuous pattern. As shown in Figure 2C, expression of nephrin and CuZn‐SOD was detected at the cell periphery in control podocytes. Interestingly, CuZn‐SOD seems localized at the front/outreach region of nephrin at the cell periphery – foot process areas. Since CuZn‐SOD is a critical antioxidant to dismutate superoxide radicals, the intimate relationship of nephrin with CuZn‐SOD or podoplanin in the foot process area suggests that CuZn‐SOD may likely exert a protective effect on slit diaphragm against oxidative insult in podocytes.

### Hypoxia downregulates nephrin and podoplanin expression, which is associated with loss of CuZn‐SOD expression in podocytes

**Figure 3:**
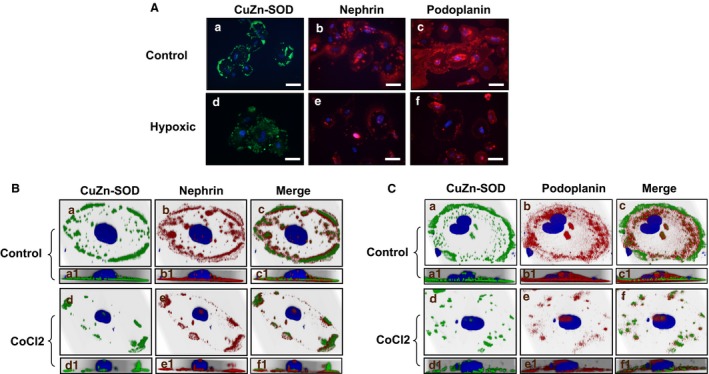
Oxidative stress downregulates CuZn‐SOD, nephrin, and podoplanin expression in podocytes. Oxidative stress was induced by two conditions: (1) cells were cultured under 2%O_2_; and (2) cells were treated with cobalt chloride, a hypoxic mimicking agent. (A) Podocytes were cultured under 2%O_2_/5%CO_2_/93%N_2_ vs. 5%CO_2_/air, a–c: cells were cultured under 5%CO_2_/air; and d–f: cells were cultured under 2%O_2_. Expression of CuZn‐SOD, nephrin, and podoplanin were all reduced in podocytes cultured at 2%O_2_ condition. Bar = 100 *μ*m. (B) and (C) Three‐dimensional reconstruction of *Z*‐stacking images of dual immunofluorescent staining of nephrin with CuZn‐SOD (B) and podoplanin with CuZn‐SOD (C) in podocytes treated with cobalt chloride compared to untreated control cells. The reconstructed 3‐dimensional images are viewed at two angles, (1) oblique, a–f series, and (2) lateral, a1‐f1 series. In control cells, both CuZn‐SOD and nephrin are located at the basal sides of cell periphery region (a1–c1), while upon treatment of cobalt chloride, there is significant reduction of both CuZn‐SOD and nephrin at the basal side of the cell (d1–f1). Cobalt chloride treatment causes significant loss of podoplanin in conjunction with the reduction of CuZnSOD compared with the control podocytes (C). AB 8/13 podocytes were used and these representative images were from at least 5–6 independent experiments.

Expression of CuZn‐SOD was reduced or lost in shed podocytes from preeclampsia (Fig. 2A and B). To determine if downregulation of nephrin and podoplanin expression seen in shed podocytes from preeclampsia is associated with reduced CuZn‐SOD expression, effects of oxidative stress on expression and distribution of CuZn‐SOD, nephrin, and podoplanin were then examined. Two strategies were undertaken: (1) podocytes were cultured under 2%O_2_/5%CO_2_/93%N_2_ compared to cells cultured under 5%CO_2_/air and (2) podocytes were treated with hypoxic inducing agent cobalt chloride. Consistent results were obtained in both experimental settings. Reduced nephrin and podoplanin expression was associated with downregulation of CuZn‐SOD expression in podocytes cultured under lowered oxygen condition (Fig. 3A) and treated with cobalt chloride (Fig. 3B and C) compared to untreated control cells.

**Figure 4:**
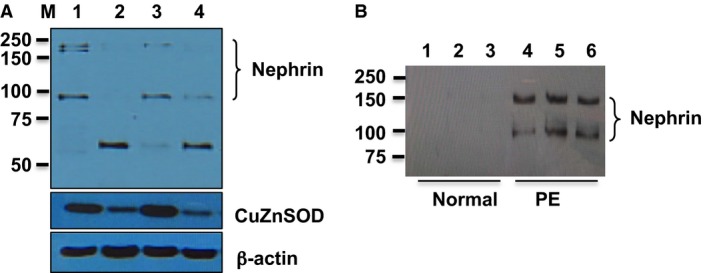
Nephrin expression in podocytes cultured under hypoxic condition and detected in urine specimen from women with preeclampsia. (A) Effects of hypoxia on podocyte nephrin and CuZn‐SOD expression. Differentiated podocytes were cultured under 2%O_2_/5%CO_2_/93%N_2_ versus 5%CO_2_/air. Total protein was extracted and subject to SDS‐PAGE. Lane 1 and 3: control cells cultured under 5%CO_2_/air; and lane 2 and 4: cells cultured under 2%O_2_. Two bands, one at 185–200 kDa and one at 90–100 kDa, were detected in control cells (lane 1 and 3). In comparison, a weak band at 90–100 kDa and a lower molecular weight fragment around 55–60 kDa was detected in cells cultured with 2%O_2_. CuZn‐SOD expression was downregulated in cells cultured under 2%O_2_. (B) Detection of soluble nephrin expression in urine specimen from preeclamptic, but not from normal, pregnancies. Nephrin was immunoprecipitated and immunoblotted. Lanes 1–3: normal pregnancies, and lanes 4–6: preeclampsia. Two molecular weight bands 150 kDa and 90–100 kDa were detected in urine specimen from women with preeclampsia.

Effects of oxidative stress mediated downregulation of nephrin and CuZn‐SOD expression were further confirmed by detection of protein expression through Western blot. Figure 4A shows podocyte nephrin expression in two representative independent experiments in which cells were cultured with 5%CO_2_/air versus 2%O_2_. Two molecular weight bands were detected for nephrin in cells cultured with 5%CO_2_/air, one approximately at 185–200 kDa and one around at 90–100 kDa. However, the band at 185–200 kDa was lost and a weak 90–100 kDa band was detected in cells cultured with 2%O_2_. Interestingly, a band at about 55–60 kDa was also detected in cells cultured under 2%O_2_ condition, but not in cells cultured with 5%CO_2_/air. CuZn‐SOD expression was also downregulated in podocytes cultured under 2%O_2_ compared to cells cultured under 5%CO_2_/air. These results provided further evidence in which oxidative stress induced downregulation of nephrin expression is associated with reduced CuZn‐SOD expression in podocytes.

### Nephrin shedding in preeclampsia

Figure 4B shows nephrin expression by immunoprecipitation and immunoblotting in urine specimen from three normal and three preeclamptic pregnant women. Nephrin was detected in specimens from preeclamptic pregnant women but not in normal pregnant women. Two bands were detected in urine specimens from preeclamptic pregnant women, one approximately at 150 kDa and one at about 90–100 kDa. Intriguingly, the 90–100 kDa band detected in the urine specimens correspond to the band detected in podocytes cultured under normoxic (5%CO_2_/air) condition. These results suggest that shed nephrin could be detected in the urinary specimen in women with preeclampsia.

## Discussion

Podocytes are a critical component of the glomerular barrier structure. Podocyte shedding is a sign of glomerular barrier injury (Karumanchi and Lindheimer 2007). We recently found that the amount of podocyte shedding is associated with the severity of preeclampsia (Wang et al. 2015). We also found that loss of podocyte nephrin expression is linked to reduced anionic glycoprotein podoplanin and altered polarity proteins PARD‐3 and PARD‐6 expression and distribution in shed podocytes from women with preeclampsia (Zhao et al. 2011). Again, in the present study podocytes were detected in all preeclamptic patients. Although the number of subjects studied is small, the fact that shed podocytes were found in urinary specimen from all preeclamptic patients, but not from normal pregnant women, is significant and further demonstrated podocyte shedding in preeclampsia. Our results are consistent with what was previously reported by Garovic et al. (2007b) (Craici et al. 2013). To investigate the potential mechanism of podocyte injury in preeclampsia, we specifically examined the association of antioxidant CuZn‐SOD with nephrin and podoplanin in shed podocytes and tested our hypothesis that increased oxidative stress contributes to podocyte injury in this pregnancy disorder.

Nephrin is considered the backbone for the slit diaphragm (Tryggvason et al. 2006). It is a type I membrane protein with a large extracellular domain and a short intracellular tail. The extracellular domain is extensively glycosylated. Appropriate localization of nephrin at the foot process is essential for the filtration slit structure and podocyte integrity (Yan et al. 2002). The renal barrier is disturbed if nephrin is reduced or displaced from the slit area. For example, nephrin was found to be redistributed to the podocyte surface in glomerular proteinuric diseases such as minimal change disease (Doublier et al. 2001). Absolute absence of nephrin from slit diaphragms produces massive proteinuria in congenital nephrotic syndrome of Finnish type (Kestilä et al. 1998). Altered nephrin expression has also been demonstrated in several glomerular‐associated diseases including diabetic nephropathy (Jim et al. 2012). However, it remains largely unknown how this integral slit protein nephrin becomes dislocated/redistributed/reduced in acquired glomerular disorders.

In this study, we found that CuZn‐SOD seems localized at the out front of the foot process area. The intimate relationship of nephrin and CuZn‐SOD found in our study suggests that CuZn‐SOD may protect slit protein from oxidative injury. On the other hand, reduced CuZn‐SOD expression may interfere with function of the foot process slit diaphragm in podocytes. This concept was supported by our findings of downregulation of CuZn‐SOD and nephrin/podoplanin expression when podocytes were challenged under hypoxic/oxidative stress conditions.

Although we did not study the direct cause‐effect relationship between CuZn SOD function and podocyte/nephrin loss, using a low‐oxygen culture condition or cells treated with CoCl2 as a model of oxidative stress condition in human AB 8/13 podocytes we observed an interesting phenomenon that hypoxia (oxidative stress) downregulates or induces dislocation of nephrin and podoplanin from foot‐process area to cytosol in podocytes, which is associated with loss of CuZn‐SOD expression in podocytes and mimics what we found in podocytes‐derived from patients with preeclampsia. Our data suggest that reduced expression of intracellular antioxidant CuZn‐SOD might be one of the contributing factors for podocyte functional protein reduction/redistribution. This notion is supported by our immunofluorescent staining observations: (1) the intimate relationship of CuZn‐SOD and nephrin at the cell periphery of the foot process area in control podocytes, and (2) reduction/dislocation of CuZn‐SOD and nephrin expression in shed podocytes from preeclampsia and in control podocytes exposed to hypoxic or oxidative stress conditions. Increased oxidative stress could have significant impact on podocyte integrity. Reduced antioxidant activity induced by hypoxia would shift the oxidative hemostasis to oxidative stress and subsequently impair podocyte function. On the other hand, increased oxidative stress may also disrupt the podocyte glycoprotein coating. Glycoproteins including nephrin, podocalyxin, podoplanin, and glomerular epithelial protein 1 (GLEEP‐1) are extensively expressed in the slit diaphragm and/or on the cell surface of podocytes, whereas significant reduction of these glycoproteins is closely associated with increased proteinuria in preeclampsia. All of them share similar features of glycosylation in their extracellular domains, either O‐linked glycoprotein in the case of podoplanin or N‐linked glycoproteins in cases of nephrin, podocalyxin, and GLEPP‐1 (Yan et al. 2002). The integrity of these glycoproteins plays a critical role in maintaining renal barrier function through their negative charges. We noticed intracellular localization of nephrin in shed podocytes from preeclampsia. Although, in this study we did not investigate how nephrin protein is synthesized or translocated from slit to cytosol or vise versa, a study by Yan et al. (2002) provided a reasonable explanation, in which these investigators found that the shortage of oxygen supply under the hypoxic treatment could lead to reduced ATP generation. During nephrin synthesis, N‐linked oligosaccharides would be added to the newly synthesized nephrin in the endoplasmic reticulum and undergo proper folding for its final target at the plasma membrane (foot process). Thus, the energy deficiency due to hypoxia could interfere with chaperone machinery within the endoplasmic reticulum and lead to retention of nephrin within the cytoplasm instead of shifting to plasma membrane of the slit diaphragm (Yang et al. 1996; Nakajo et al. 2007). In fact, hemoglobin/free heme associated with increased oxidative stress has been reported to contribute podocyte injury associated with preeclampsia (Gilani et al. 2017).

Increased oxidative stress induced downregulation of nephrin expression and nephrin shedding in preeclampsia was further demonstrated by nephrin expression detected by western blot (Fig. 4A) and by immunoprecipitation/immunoblotting of nephrin in the urinary specimen from women with preeclampsia (Fig. 4B). Two molecular weight bands 180–200 kD and 90–100 kD were detected in the control podocytes and two molecular weight bands 150 kD and 90–100 kD were detected in the urinary specimen from women with preeclampsia. Although we did not determine the relationship between 180 and 200 kD and 150 kD nephrin detected in the podocytes and urinary specimen, the 180–200 kD nephrin was most likely the mature form of nephrin and the 150 kD nephrin could be the deglycosylated one (Yan et al. 2002; Khoshnoodi et al. 2003). Interestingly, the molecular weight fragment of 90–100 kDa nephrin protein detected by immunoprecipitation/immunoblotting in urine specimen from preeclamptic patients was corresponded to nephrin band detected in podocytes that were cultured under hypoxic condition. These findings provide plausible evidence that oxidative stress is a contributing factor that induces podocyte injury, podocyturia, and/or podocyte protein shedding in preeclampsia. Since all urine samples were centrifuged to remove shed podocytes before immunoprecipitation, we believed that nephrin detected in the urine samples was shed soluble form from podocytes, not from shed podocytes. In addition, we also detected a smaller molecular weight protein about 55–60 kDa in hypoxic‐treated podocytes. At this time we do not know what the nature is for the 55–60 kDa nephrin detected by western blotting in cells cultured under oxidative stress, a similar 55–60 kDa nephrin band was detected by (Khoshnoodi et al. (2007), in which terminal sugar residues of N‐linked nephrin glycans were identified when digoxygenin‐labeled lectins were used.

In summary, our previous study revealed increased nitrotyrosine staining and decreased CuZn‐SOD staining in kidney biopsy tissues from women who had preeclampsia (Zhao et al. 2009), suggesting increased oxidative stress in kidneys in preeclampsia. In the present study, we found that reduced nephrin and podoplanin expression is associated with loss of CuZn–SOD expression in shed podocytes from preeclamptic patients. We further found that increased oxidative stress could induce podocyte nephrin shedding and cellular dislocation, which corresponds to shed nephrin detected in the patient urine specimen. Thus, our results provide plausible evidence that increased oxidative stress is a contributing factor of podocyte injury and podocyte protein shedding in preeclampsia.

## Conflicts of Interest

The authors declare no conflicts of interest.

## References

[phy213785-bib-0001] Aita, K. , M. Etoh , H. Hamada , C. Yokoyama , A. Takahashi , T. Suzuki , et al. 2009 Acute and transient podocyte loss and proteinuria in preeclampsia. Nephron Clin. Pract. 112:c65–c70. 19390204 10.1159/000213083

[phy213785-bib-0002] Craici, I. M. , S. J. Wagner , K. R. Bailey , P. D. Fitz‐Gibbon , C. M. Wood‐Wentz , S. T. Turner , et al. 2013 Podocyturia predates proteinuria and clinical features of preeclampsia: longitudinal prospective study. Hypertension 61:1289–1296. 23529165 10.1161/HYPERTENSIONAHA.113.01115 PMC3713793

[phy213785-bib-0003] Dai, Z. J. , J. Gao , X. B. Ma , K. Yan , X. X. Liu , H. F. Kang , et al. 2012 Up‐regulation of hypoxia inducible factor‐1*α* by cobalt chloride correlates with proliferation and apoptosis in PC‐2 cells. J. Exp. Clin. Cancer Res. 31:28. 22453051 10.1186/1756-9966-31-28 PMC3359273

[phy213785-bib-0004] Doublier, S. , V. Ruotsalainen , G. Salvidio , E. Lupia , L. Biancone , P. G. Conaldi , et al. 2001 Nephrin redistribution on podocytes is a potential mechanism for proteinuria in patients with primary acquired nephrotic syndrome. Am. J. Pathol. 158:1723–1731. 11337370 10.1016/S0002-9440(10)64128-4 PMC1891937

[phy213785-bib-0005] Garovic, V. D. , S. J. Wagner , L. M. Petrovic , C. E. Gray , P. Hall , H. Sugimoto , et al. 2007a Glomerular expression of nephrin and synaptopodin, but not podocin, is decreased in kidney sections from women with preeclampsia. Nephrol. Dial. Transplant. 22:1136–1143. 17255128 10.1093/ndt/gfl711

[phy213785-bib-0006] Garovic, V. D. , S. J. Wagner , S. T. Turner , D. W. Rosenthal , W. J. Watson , B. C. Brost , et al. 2007b Urinary podocyte excretion as a marker for preeclampsia. Am. J. Obstet. Gynecol. 196: 320e321–320e327. 17403404 10.1016/j.ajog.2007.02.007

[phy213785-bib-0007] Gilani, S. I. , U. D. Anderson , M. Jayachandran , T. L. Weissgerber , L. Zand , W. M. White , et al. 2017 Urinary Extracellular Vesicles of Podocyte Origin and Renal Injury in Preeclampsia. J. Am. Soc. Nephrol. 28:3363–3372. 28729288 10.1681/ASN.2016111202 PMC5661277

[phy213785-bib-0008] Goldberg, M. A. , and T. J. Schneider . 1994 Similarities between the oxygen‐sensing mechanisms regulating the expression of vascular endothelial growth factor and erythropoietin. J. Biol. Chem. 269:4355–4359. 8308005

[phy213785-bib-0009] Greiber, S. , T. Münzel , S. Kästner , B. Müller , P. Schollmeyer , and H. Pavenstädt . 1998 NAD(P)H oxidase activity in cultured human podocytes: effects of adenosine triphosphate. Kidney Int. 53:654–663. 9507211 10.1046/j.1523-1755.1998.00796.x

[phy213785-bib-0010] Jim, B. , M. Ghanta , A. Qipo , Y. Fan , P. Y. Chuang , H. W. Cohen , et al. 2012 Dysregulated nephrin in diabetic nephropathy of type 2 diabetes: a cross sectional study. PLoS ONE 7:e36041. 22615747 10.1371/journal.pone.0036041 PMC3355157

[phy213785-bib-0011] Karumanchi, S. A. , and M. D. Lindheimer . 2007 Preeclampsia and the kidney: footprints in the urine. Am. J. Obstet. Gynecol. 196:287–288. 17403396 10.1016/j.ajog.2007.02.013 PMC2121665

[phy213785-bib-0012] Kestilä, M. , U. Lenkkeri , M. Männikkö , J. Lamerdin , P. McCready , H. Putaala , et al. 1998 Positionally cloned gene for a novel glomerular protein–nephrin–is mutated in congenital nephrotic syndrome. Mol. Cell 1:575–582. 9660941 10.1016/s1097-2765(00)80057-x

[phy213785-bib-0013] Khoshnoodi, J. , K. Sigmundsson , L. G. Ofverstedt , U. Skoglund , B. Obrink , J. Wartiovaara , et al. 2003 Nephrin promotes cell‐cell adhesion through homophilic interactions. Am. J. Pathol. 163:2337–2346. 14633607 10.1016/S0002-9440(10)63590-0 PMC1892394

[phy213785-bib-0014] Khoshnoodi, J. , S. Hill , K. Tryggvason , B. Hudson , and D. B. Friedman . 2007 Identification of N‐linked glycosylation sites in human nephrin using mass spectrometry. J. Mass Spectrom. 42:370–379. 17212372 10.1002/jms.1170

[phy213785-bib-0015] Ma, R. , Y. Gu , L. J. Groome , and Y. Wang . 2011 ADAM17 regulates TNF*α* production by placental trophoblasts. Placenta 32:975–980. 22018416 10.1016/j.placenta.2011.09.015 PMC3360543

[phy213785-bib-0016] Maxwell, P. H. , M. S. Wiesener , G. W. Chang , S. C. Clifford , E. C. Vaux , M. E. Cockman , et al. 1999 The tumour suppressor protein VHL targets hypoxia‐inducible factors for oxygen‐dependent proteolysis. Nature 399:271–275. 10353251 10.1038/20459

[phy213785-bib-0017] Nakajo, A. , J. Khoshnoodi , H. Takenaka , E. Hagiwara , T. Watanabe , H. Kawakami , et al. 2007 Mizoribine corrects defective nephrin biogenesis by restoring intracellular energy balance. J. Am. Soc. Nephrol. 18:2554–2564. 17687078 10.1681/ASN.2006070732

[phy213785-bib-0018] O'Hare, M. J. , J. Bond , C. Clarke , Y. Takeuchi , A. J. Atherton , C. Berry , et al. 2001 Conditional immortalization of freshly isolated human mammary fibroblasts and endothelial cells. Proc. Natl Acad. Sci. USA 98:646–651. 11209060 10.1073/pnas.98.2.646 PMC14642

[phy213785-bib-0019] Saleem, M. A. , M. J. O'Hare , J. Reiser , R. J. Coward , C. D. Inward , T. Farren , et al. 2002 A conditionally immortalized human podocyte cell line demonstrating nephrin and podocin expression. J. Am. Soc. Nephrol. 13:630–638. 11856766 10.1681/ASN.V133630

[phy213785-bib-0020] Spargo, B. , C. P. McCartney , and R. Winemiller . 1959 Glomerular capillary endotheliosis in toxemia of pregnancy. Arch. Pathol. 68:593–599. 13833162

[phy213785-bib-0021] Tryggvason, K. , J. Patrakka , and J. Wartiovaara . 2006 Hereditary proteinuria syndromes and mechanisms of proteinuria. N. Engl. J. Med. 354:1387–1401. 16571882 10.1056/NEJMra052131

[phy213785-bib-0022] Wang, Y. , S. Zhao , S. Loyd , and L. J. Groome . 2012 Increased urinary excretion of nephrin, podocalyxin, and *β*ig‐h3 in women with preeclampsia. Am. J. Physiol. Renal Physiol. 302:F1084–F1089. 22301621 10.1152/ajprenal.00597.2011

[phy213785-bib-0023] Wang, Y. , Y. Gu , S. Loyd , X. Jia , and L. J. Groome . 2015 Increased urinary levels of podocyte glycoproteins, matrix metallopeptidases, inflammatory cytokines, and kidney injury biomarkers in women with preeclampsia. Am. J. Physiol. Renal Physiol. 309:F1009–F1017. 26671966 10.1152/ajprenal.00257.2015

[phy213785-bib-0024] Yan, K. , J. Khoshnoodi , V. Ruotsalainen , and K. Tryggvason . 2002 N‐linked glycosylation is critical for the plasma membrane localization of nephrin. J. Am. Soc. Nephrol. 13:1385–1390. 11961028 10.1097/01.asn.0000013297.11876.5b

[phy213785-bib-0025] Yang, D. H. , M. Goyal , K. Sharif , D. Kershaw , P. Thomas , R. Dysko , et al. 1996 Glomerular epithelial protein 1 and podocalyxin‐like protein 1 in inflammatory glomerular disease (crescentic nephritis) in rabbit and man. Lab. Invest. 74:571–584. 8600307

[phy213785-bib-0026] Zhao, S. , X. Gu , L. J. Groome , and Y. Wang . 2009 Decreased nephrin and GLEPP‐1, but increased VEGF, Flt‐1, and nitrotyrosine, expressions in kidney tissue sections from women with preeclampsia. Reprod. Sci. 16:970–979. 19528353 10.1177/1933719109338630 PMC3065976

[phy213785-bib-0027] Zhao, S. , Y. Gu , G. Coates , L. J. Groome , M. A. Saleem , P. W. Mathieson , et al. 2011 Altered nephrin and podoplanin distribution is associated with disturbed polarity protein PARD‐3 and PARD‐6 expressions in podocytes from preeclampsia. Reprod. Sci. 18:772–780. 21422051 10.1177/1933719111398145

